# Water quality assessment of Australian ports using water quality evaluation indices

**DOI:** 10.1371/journal.pone.0189284

**Published:** 2017-12-15

**Authors:** Sayka Jahan, Vladimir Strezov

**Affiliations:** Department of Environmental Sciences, Faculty of Science and Engineering, Macquarie University NSW, Sydney, Australia; Jinling Institute of Technology, CHINA

## Abstract

Australian ports serve diverse and extensive activities, such as shipping, tourism and fisheries, which may all impact the quality of port water. In this work water quality monitoring at different ports using a range of water quality evaluation indices was applied to assess the port water quality. Seawater samples at 30 stations in the year 2016–2017 from six ports in NSW, Australia, namely Port Jackson, Botany, Kembla, Newcastle, Yamba and Eden, were investigated to determine the physicochemical and biological variables that affect the port water quality. The large datasets obtained were designed to determine the Water Quality Index, Heavy metal Evaluation Index, Contamination Index and newly developed Environmental Water Quality Index. The study revealed medium water quality index and high and medium heavy metal evaluation index at three of the study ports and high contamination index in almost all study ports. Low level dissolved oxygen and higher level of total dissolved solids, turbidity, fecal coliforms, copper, iron, lead, zinc, manganese, cadmium and cobalt are mainly responsible for the poor water qualities of the port areas. Good water quality at the background samples indicated that various port activities are the likely cause for poor water quality inside the port area.

## Introduction

Large number of seaports are situated along the coastal belt of Australia that are engaged with different commercial activities, such as transportation of passengers, livestock, coal, iron ore, steel and different business products [1, 2]. Regardless of their size, the environmental impact of seaports largely depends on these commercial activities [3]. Seaports are very complex systems with a wide range of environmental particulars, including releases to water, air and soil, waste production, noise and dredging, amongst others [4]. In port areas or in their vicinity, several activities, such as fisheries, industrial installations, storage of hazardous materials, may cause further environmental impacts. Deballasting of waters from ships has been shown to impact distribution of pollutants and pathogens with adverse health and environmental impacts [5]. Finally, the continuous movement of ships in a confined area and the intense traffic increase the frequency of accidents often causing risk of release of hazardous materials in the port area [6].

The ecological sustainability and biological productivity of coastal and marine ecosystems largely depend on the coastal water quality. The coastal regions are believed to have richer biodiversity than the open ocean regions [7]. However, the coastal marine water quality is declining continuously due to the elevated concentration of various pollutants, among which total dissolved and suspended solids, nutrient and organic compounds [8] often cause turbidity [9] and significant reduction in dissolved oxygen levels [10]. The distribution of trace metal pollutants do not have direct impact on the optical properties of water, however, their presence influences the storage properties of water, viz., pH, temperature, total dissolved solid and turbidity [11]. Although some pollutants (trace metals, biological and nutrients) occur naturally in the environment, elevated pollutant concentrations in the coastal port areas are generally the consequence of effluent discharge from shipping activities, cargo handling, container loading and storage, and vehicle marshalling, urban storm water and agricultural and industrial run-off [12–14]. There is an abundant volume of work that investigates concentration of pollutants in sediments around various ports in Australia. However, there is only limited work published on the pollutants present and dissolved in the water. Jonathan et al. [15] suggest that water profile preserves the pollution sequence in a more reliable way, and further states that beach water quality deteriorates more than the sediments. Although sea ports act as a major industrial activity and central part of the land-sea interface in the coastal zone of Australia, relatively little attention has been given to these areas, where different shipping operations may have considerable impacts on the port environment [16].

The aim of this study was to generate the most reliable and large data for water quality and trace metal concentrations in Australian ports. The study was designed to obtain Water Quality Index (WQI), Heavy metal Pollution Index (HEI), Contamination Index (C_d_) and Environmental Water Quality Index (EWQI) to present the large complex datasets in a more comprehensive and understandable approach. This study also considered the significance of tides on the distribution of pollutants and impacts of different port activities on the water environment. Moreover, in this study, analysis of variance (F-test) was used to determine the similarities or dissimilarities between sampling sites and correlation among the physicochemical parameters and heavy metals were also analysed to determine the degree of dependency of the parameters.

## Materials and methods

### Study site

**Fig 1:**
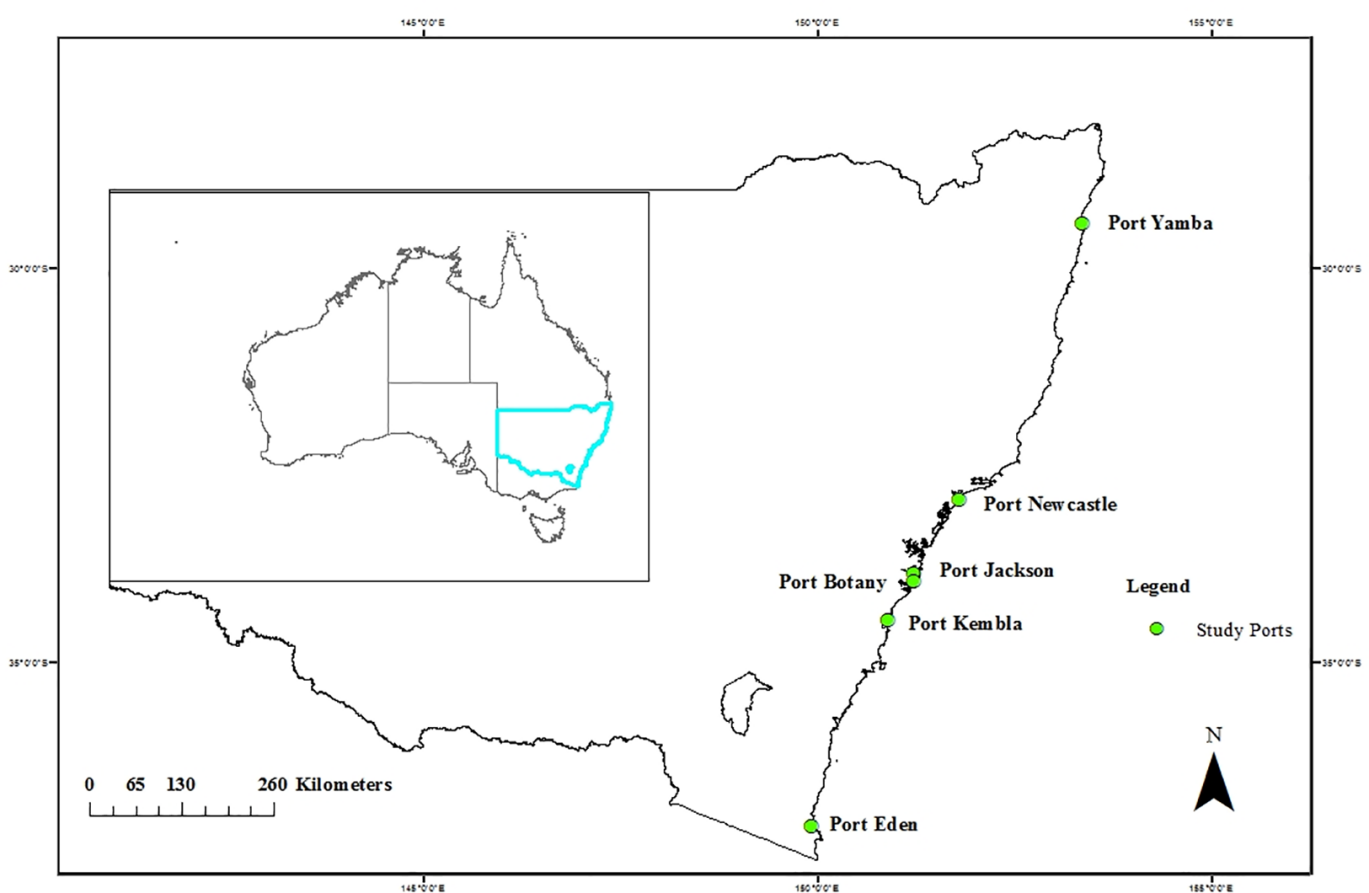
Map of the study are showing study ports.

New South Wales, which is economically the most important state in Australia, has a number of sea ports, out of which Port Jackson, Botany, Newcastle, Kembla, Eden and Yamba are the largest commercial ports. The sampling localities in this study were all the six important ports of New South Wales, Australia which are away from one another and are engaged with different shipping activities. Port Jackson of Sydney Harbour is engaged with passenger shipping, recreational boating and water sports [17] and is generally a well mixed estuary [18] because of low freshwater discharge and tidal turbulence [19]. Port Botany, located in the mouth of George river, is now the site of Sydney's two major stevedoring and bulk liquid facilities. Container, cruide oil and bulk liquid operations (fossil fuel, chemical and bio-fuel) are the major activities of Port Botany [20]. Port Kembla Harbour is a major export location for coal mined in the southern and western regions of New South Wales with many facilities and berths including the grain terminal, bulk liquids, oil, various products berths (steel berth) and multi-purpose berths (fertiliser, pulp & steel products). Moreover, the port is important for importing iron ore, dolomite, limestone, sulphur, copper, phosphate rock and petroleum products and exporting iron and steel, coal, coke, tinplate and copper cables [20]. The Port of Newcastle is the world's largest coal export port that also deals with raw materials for steelworks, fertiliser and aluminium industries, grain, steel products, mineral sands and woodchips [20] and is known as one of Australia's largest ports by throughput tonnage [16]. Port Yamba is Australia's eastern most sea port is the home of the New South Wales’ second largest fishing fleet and handles a range of imports and exports, such as container liquid berth-livestock and explosive products. The Port of Eden is a small seaport, located in the South Coast) region of New South Wales, is one of the largest fishing fleets in New South Wales, Australia. Woodchip export is currently the major trade for the port, while the principal imports are break bulk and machinery and equipment, mainly for the oil and gas industry [20]. The map of the study area and coordinates details of the sampling location points are listed in Fig 1 and S1 Table.

### Sampling procedure and analysis

The port water sampling was carried out during the period of December 2016 to March 2017 during the period of high and low tides. From each port, five water samples were collected from different points among which one was background sample collected from outside of the port area. The sampling positions were recorded by a GPS. Composite water sample was prepared from each point by mixing water from different depths, which was collected using Niskin water sampler. The Niskin water sampler was previously cleaned with deionised water and conditioned for at least 15 minutes at each depth of water collection.

Samples were collected in clean screw capped polypropylene bottles without any preservatives and preserved below 4°C. On-site measurement of pH, dissolved oxygen (DO) and temperature were performed using EUTECH EcoScan pH6, EUTECH CyberScan DO 300 meter. The turbidity and conductivity were measured using HANNA HI 98703 turbidimeter and EUTECH CyberScan CON 400 conductivity meter. All instruments were calibrated prior to each sampling day. Five replicated measurements were taken for the quality purpose on each sampling site. Fecal coliform was analysed following the USEPA approved IDEXX Laboratories Colilert test kit procedure [21]. The National Association of Testing Authorities (NATA) and Australian accredited Envirolab Services analysed all the remaining inorganic, organic and standard water quality parameters [22]. The samples were filtered in laboratory before testing for the dissolved parameters in compliance to the approved methods. For trace metal analysis samples were acidified with nitric acid to <2 pH [23], and then samples and blank were analyzed for silver, aluminum, arsenic, cadmium, chromium, copper, iron, manganese, nickel, lead, selenium, zinc, mercury, boron, cobalt, molybdenum and tin using Inductively Coupled Plasma–Atomic Emission Spectrometry (ICP-AES) and inductively coupled plasma-mass spectrometry (ICP-MS, Optima 2100 DV ICP System, Perkin Elmer). Quality control was performed in accordance with NATA (National Association of Testing Authorities) guidelines for method validation [24] and measurement uncertainty [25] by analysing certified reference material AC-E with a composition of 14.75% Al_2_O_3_; 2.54% Fe_2_O_3_ and 0.06% MnO_2_. The recoveries were 100.9% for Al, 93.1% for Mn and 98.2% for Fe.

### Water quality assessment

Sample water quality was assessed and quality indices were calculated as outlined below.

#### Water quality index (WQI) calculation

Water Quality Index (WQI) expresses the overall water quality of a particular source at a certain time using a ‘single value’ based on selected water quality variables [26]. The WQI incorporates nine parameters including temperature, dissolved oxygen, pH, phosphate, nitrate, total dissolved solids, biological oxygen demand (BOD) and fecal coliform [27]. The index is calculated from Q value and weight factor W, where Q indicates the level of water quality relative to any single parameter and the weight factor represents the relative importance of the single parameter to the overall water quality. The overall water quality ranking criteria falls under five categories which are very bad when WQI is < 25, bad when WQI is 26–50, moderate when WQI is 51–70, good when WQI is 71–90 and very good when in the range of 91–100 [28,29].

$$WQI=\sum W_{i}Q_{i}=W_{Temperature}Q_{Temperature}+W_{DO}Q_{DO}+W_{pH}Q_{pH}+W_{Nitrate}Q_{Nitrate}+W_{Turbidity}Q_{Turbidity}+W_{TDS}Q_{TDS}+W_{Phosphate}Q_{Phosphate}+W_{BOD}Q_{BOD}+W_{Fecal\;Coliform}Q_{Fecal\;Coliform}$$

#### Contamination index (C_d_)

The Contamination index (C_d_) was calculated separately for each analysed sample of water, as a sum of the contamination factors of individual components exceeding the upper permissible value. The index developed by Backman et al. (1998) and Prasanna et al. (2012) relates the quality of water to human health risk and is calculated as:

$$C_{d}=\sum_{i=1}^{n}C_{fi}$$

Where $C_{fi}=\frac{C_{Ai}}{C_{Ni}}-1$; C_fi_, C_Ai_ and C_Ni_ represents contamination factor, analytical value and upper permissible concentration of the i^th^ component respectively and N denotes normative value [30, 31] and hence C_Ni_ is taken as maximum allowable concentration (MAC).

The calculated values are grouped into low (C_d_ <1), medium (C_d_ = 1–3) and high (C_d_ >3) contamination [32].

#### Heavy metal evaluation index (HEI)

HEI describes water quality condition in response to anthropogenic heavy metals and is calculated by [31]:

$$HEI=\sum_{i=1}^{n}\frac{H_{c}}{H_{mac}}$$

Where, H_c_ is monitored value and H_mac_ is maximum admissible concentration of the i^th^ parameter.

The HEI values are grouped into low contamination (HEI< 400), Medium concentration (HEI = 400–800) and high contamination (HEI > 800) [31].

#### Environmental assessment

The environmental assessment was performed by comparing the measured concentrations of the toxic elements with the available trigger values recommended by Australian and New Zealand Environment and Conservation Council (ANZECC) guidelines for marine water and with internationally published guidelines including United States Environmental Protection Agency (USEPA) and United Kingdom Department for Environment, Food and Rural Affairs. All the comparative standard guidelines [33–35] are listed in S2 Table.

#### Environmental water quality index

The environmental water quality assessment index is a newly proposed index by [36] which is calculated by multiplying the concentration of each contaminant measured in the water samples with the corresponding hazard intensity to determine the water quality impact. The hazard intensity of each parameter was determined according to the total score assigned by the Toxicological Profiles of the Priority List of Hazardous Substances prepared by the Agency for Toxic Substances and Disease Registry (ATSDR), the Division of Toxicology and Environmental Medicine, Atlanta, USA [37]. The total score of each trace element was multiplied by its analysed concentration and products were added to calculate the trace element toxicity index (TETI). The environmental water quality index (EWQI) was calculated by dividing the water quality index (WQI) by the trace element toxicity index (TETI).

$$\mathrm{Environmental}\;\mathrm{water}\;\mathrm{quality}\;\mathrm{index}\;EWQI=\frac{wQI}{\sum_{\dot{i}=1}^{n}C_{i}*Ts_{i}}$$

Where, WQI = water quality index; C_i_ = Concentration of individual trace element; TS_i_ = Total Score (Agency for Toxic Substances and Disease Registry) of individual trace element.

## Results and discussion

The descriptive statistics of the eight physicochemical and one biological parameters of water quality with their observed standard deviations for each site were calculated as shown in Table 1, and used to assess the quality of port water. The original data of the measurements are available in S3 Table. The pH values in five out of the six ports ranged from 7.6 to 8.3, which are within the standard values, except for port Eden where the water pH ranged from 6.92 to 7.89 and was not affected significantly by the tides. The Dissolved Oxygen (DO %) values for most of the water samples were markedly lower during high tide, except for Port Eden where DO was significantly affected by the low tide. Most of the background samples had higher DO levels than the port water samples and, out of six ports; three ports (Port Jackson, Port Newcastle and Port Yamba) had standard levels of DO according to ANZECC guidelines [33]. The other three ports (Port Botany, Kembla and Eden) had DO levels lower than the standard guidelines. At Port Eden the DO was significantly low (36.1, 52.3, and 23.6) at three points inside the port area during low tide due to intensive commercial fishing activities and hydrocarbon contamination from imported petroleum products inside the main port [38]. Total Dissolved Solids (TDS) of the port water were significantly affected by the tide, showing higher values during low tide comparing to the high tide for the same site. The water in all ports, except for Port Kembla and Newcastle, had higher turbidity values than the background water, which indicates impact of port activities on the water environment. Almost all ports had standard BOD_5_ levels, except for Port Botany and Port Eden. The three major sampling points at Port Eden had very high BOD_5_ levels. According to Pollard and Rankin [38] Port Botany and Eden are moderately polluted by organic waste. Moreover, presence of fecal coliforms in the port area is detected during both high and low tides. Port Jackson, Botany, Kembla and Eden were positively affected by high tide, whereas low tide increased the fecal coliforms in the water at port Newcastle and Yamba. Among all ports, the amount of fecal coliforms was significantly higher in the water at port Jackson, Yamba and Eden than the corresponding background samples, which clearly indicates the impact of cruise ships, fishing fleet and recreational boating on the port environment.

**Table 1:** Water quality index parameters.

Parameter			pH	DO %	TDS mg/l	Turbidity NTU	Temp °C	Phosphate mg/l	Nitrate mg/l	BOD mg/l	FC /100ml	Conductivity ms	WQI	Water Quality
**Study Area**	Tide	Weight Factor	0.11	0.17	0.07	0.08	0.1	0.1	0.1	0.11	0.16			
**Port Jackson**	H		7.75 ± 0.095	75 ± 8.72	43393 ± 953.66	0.505 ± 0.15	23.3	0.009 ±0.0012	0.035 ± 0.014	0.73 ± 0.25	212 ± 201	51 ± 1.65	75.78 ± 3.11	GGGG
	L		7.66 ± 0.064	81.4 ± 2.68	49325 ± 12604	0.63 ± 0.009	25.4	0.011 ± 0.0015	0.037 ± 0.012	0.57 ± .096	6 ± 5.55	49 ± 0.85	80.33 ± 7.2	GGGG
	B		7.70 ± 0.007	85 ± 7.07	45450 ± 6764.18	0.53 ± 0.25	24.4	0.013 ± 0.0028	0.034 ± 0.03	0.65 ± 0.070	3.1 ± 4.38	51 ± 0.64	81.1 ± 9.12	GG
**Port Botany**	H		7.64 ± 0.78	67.18 ± 6.04	46120 ± 4093	2 ± 1.41	24	0.0084 ± 0.0024	0.072 ± 0.107	1.1 ± 0.25	410 ± 428.10	49 ± 0.45	73.21 ± 2.42	GGGG
	L		7.65 ± 0.017	70.78 ± 1.56	45690 ± 5445	2 ± 0.59	26.1	0.0097 ± 0.002	0.066 ± 0.003	2 ± 0.95	287 ± 482.25	50 ± 0.43	76.24 ± 5.71	MGGG
	B		7.72 ± 0.035	70.50 ± 0.78	42965 ± 1477.85	0.27 ± 0.064	25	0.0048 ± 0	0.0315 ± 0.006	0.8 ± 0.28	502 ± 709.23	51 ± 0.28	68.91 ± 1.57	GG
**Port Kembla**	H		7.88 ± 0.25	75 ± 6.98	40720 ± 818.10	0.29 ± 0.105	22.8	0.010 ± 0.004	0.014 ± 0.014	1 ± 0.13	507 ± 375.72	52 ± 0.32	74.93 ± 2.89	GGGG
	L		7.9 ± 0.028	76.87 ± 5.27	41219 ± 363.87	0.19 ± 0.015	23	0.010 ± 0.004	0.010 ± 0.011	1 ± 0.44	35 ± 41.71	52 ± 0.20	80.20 ± 2.7	GGGG
	B		7.70 ± 0.25	76 ± 7.63	40600 ± 1357.65	0.44 ± 0.17	22.6	0.013 ± 0.002	0.0048 ± 0	0.48 ± 0.002	401 ± 176.09	52.3 ± 0.14	75.21 ± 1.14	GG
**Port Newcastle**	H		7.8 ± 0.23	82.08 ± 5.02	40335 ± 1654.36	0.74 ± 0.15	24	0.006 ± 0.0005	0.004 ± 0.0006	0.9 ± 0	672 ± 397	52 ± 0.61	74.69 ± 0.38	GGGG
	L		7.9 ± 0.02	83.45 ± 5.76	46703 ± 1352.7	0.55 ± 0.14	24.2	0.0065 ± 0.0015	0.0046 ± 0.0012	0.9 ± 0	1003 ± 0	52 ± 0.60	75.87 ± 1.84	GGGG
	B		8.11 ± 0.27	85.7 ± 6.08	42744 ± 3285	0.89 ± 0.24	24.2	0.0048 ± 0	0.005 ± .0002	1 ± 0.35	706 ± 420	52 ± 1.56	74.89 ± 3.01	GG
**Port Yamba**	H		7.7 ± 0.26	75.6 ± 3.40	85860 ± 2441	0.92 ± 0.29	22.4	0.006 ± 0.0009	0.0048 ± 0	0.9 ± 0	444 ± 511	53 ± 0	77.37 ± 4.65	GGGG
	L		7.7 ± 0.086	81 ± 3.86	74248 ± 24422	0.81 ± 0.41	22.3	0.0038 ± 0.005	0.0042 ± 0.0007	1 ± 0.31	1003 ± 0	53 ± 0	74.12 ± 0.72	GGGG
	B		8.3 ± 0.077	80.2 ± 3.4	87580 ± 17239	0.30 ± 0.042	22.4	0.005 ± 0.042	0.0049 ± 0	0.9 ± 0	25 ± 131.17	53 ± 0	81.52 ± 1.41	GG
**Port Eden**	H		7.72±0.36	87.8±4.32	67125±8822	0.1925±0.060	19.8	0.014±0.008	0.043±0.038	0.9±0	899.875±205.50	54.525±0.330	75.76±0.860	GGGG
	L		7.40±0.36	57.67±22.90	53100±14651	3.68±3.70	20.2	1.554±2.518	0.013±0.0082	89±129.038	570.125±418.13	54.85±0.822	51.98±16.47	MMMG
	B		7.60±0.36	88.5±0.424	50400±10323	0.70±0.29	20.1	0.278±0.226	0.058±0.0215	6.50±5.031	366.25±503.85	54.7±0.707	74.52±1.382	GG

H = High tide, L = Low tide, B = Background, G = Good, M = Moderate.

The WQI for the port water quality during both high and low tides were reported as good, with the exception of Port Botany and Eden, as shown in Table 1. The WQI analysis revealed that Port Botany and Eden were the two most affected sites along the entire reach of the seaports. Out of nine parameters considered for this study, DO (%) and fecal coliforms were the two deciding parameters exhibiting the maximum influence in WQI calculations (Table 1). Port Botany and Eden experienced lower DO and higher fecal coliform concentrations, thus signifying the moderate port water quality.

The impact of tides on the water quality index was tested with the statistical t-test, where P > 0.11 advocates no significant difference of the tidal conditions, as shown in Table 2. Although the t-test results presented no significant difference between the tidal conditions, some of the water quality parameters (DO, turbidity, fecal coliform) showed variations with tide, as presented in Table 1.

**Table 2:** Test of significance of tides on different physiochemical parameters.

Study Area	P -value	Pearson correlation
**Port Jackson**	0.174	0.9999
**Port Botany**	0.115	0.9999
**Port Kembla**	0.4839	0.9999
**Port Newcastle**	0.1537	0.9999
**Port Yamba**	0.1654	0.9999
**Port Eden**	0.16067	0.9999

The concentration of trace metals in the port water and the background sites are given in Table 3. The concentration of selenium, mercury, beryllium, bismuth and tin were all below the detection limits of the measuring equipment employed in the study. The mean concentrations of silver, aluminum, arsenic, nickel, vanadium, boron were all within the ANZECC and other international guidelines. The maximum concentration of copper in all the studied ports water was much higher than ANZECC guidelines and exceeded other international guidelines with the highest concentration (0.04 mg/l) in Port Yamba. Moreover, the mean concentration of copper also exceeded the ANZECC guidelines, except for Port Kembla and Newcastle. Very high concentrations of iron were found in the port water of Botany, Newcastle, Yamba and Eden though the others also exceeded the UK [35] guidelines. All background samples, except for Port Jackson, also showed high concentrations of iron. In the port water of Newcastle, Yamba and Eden the maximum concentration of manganese was higher than the background sample. The maximum concentration of lead in the water of port Botany (0.007) and Yamba (0.005) exceeded the ANZECC [33] (0.0022) guidelines but was within the UK [35] (0.025) and USA EPA [34] (0.0081) guidelines. In addition, all the background samples had much lower concentration of lead compared to the port area. According to the ANZECC guidelines, the water in all of the studied ports, except Port Kembla, contained very high concentrations of zinc, which exceeded the guidelines. However, if the EPA and UK guidelines are considered, the values were all within the standards. Furthermore, all the background samples have very low concentrations of zinc except the background sample of port Jackson. Among all ports, only the water of Port Eden contained very high concentration of cadmium and cobalt that exceeded the ANZECC guidelines, but were absent in the background water.

**Table 3:** Trace metals concentrations in sample water.

Study Area	Port Jackson				Port Botany				Port Kembla				Port Newcastle				Port Yamba				Port Eden			
**Analytes (mg/l)**	Min	Max	Mean	Bg	Min	Max	Mean	Bg	Min	Max	Mean	Bg	Min	Max	Mean	Bg	Min	Max	Mean	Bg	Min	Max	Mean	Bg
**Ag**	Bd	Bd	Bd	Bd	Bd	0.001	0.001	Bd	Bd	0.004	0.003	0.003	Bd	Bd	Bd	Bd	Bd	0.001	0.001	Bd	Bd	0.001	Bd	Bd
**Al**	0.014	0.028	0.019	0.018	0.018	0.151	0.113	0.02	0.001	0.037	0.01	0.02	0.003	0.125	0.028	0.004	0.022	1.17	0.214	0.03	0.013	0.016	0.015	Bd
**As**	0.003	0.004	0.003	0.003	0.002	0.008	0.004	0.005	0.002	0.005	0.003	0.003	0.002	0.004	0.0033	0.003	0.002	0.005	0.0029	0.002	0.002	0.006	0.0032	0.0035
**Cd**	Bd	0.001	Bd	Bd	Bd	Bd	Bd	Bd	Bd	Bd	Bd	Bd	Bd	Bd	Bd	Bd	Bd	Bd	Bd	Bd	0.001	0.007	0.001	Bd
**Cr**	Bd	0.001	0.001	Bd	0.001	0.002	0.001	Bd	Bd	0.001	0.001	Bd	Bd	0.001	0.001	Bd	0.001	0.002	0.001	Bd	Bd	0.001	Bd	Bd
**Cu**	0.003	0.004	0.0035	0.003	0.001	0.009	0.003	0.0025	Bd	0.004	0.002	Bd	Bd	0.005	0.0021	Bd	Bd	0.04	0.009	Bd	Bd	Bd	Bd	Bd
**Fe**	0.022	0.077	0.042	0.005	0.015	0.531	0.1	0.018	0.008	0.036	0.017	0.012	0.008	0.191	0.0514	0.013	0.016	0.27	0.143	0.112	Bd	0.094	0.082	0.016
**Mn**	0.002	0.004	0.003	0.003	0.002	0.006	0.005	0.0025	0.001	0.009	0.003	0.0015	0.001	0.034	0.0087	0.001	0.009	0.044	0.015	0.01	0.001	0.051	0.01	0.0045
**Ni**	Bd	0.001	0.001	Bd	Bd	0.001	0.001	Bd	Bd	Bd	Bd	Bd	Bd	Bd	Bd	Bd	Bd	Bd	Bd	Bd	Bd	0.009	0.0016	0.001
**Pb**	0.001	0.002	0.001	0.001	0.001	0.007	0.002	0.001	Bd	0.001	0.001	Bd	Bd	0.001	0.001	Bd	Bd	0.005	0.0008	Bd	Bd	0.001	Bd	Bd
**Se**	Bd	Bd	Bd	Bd	Bd	Bd	Bd	Bd	Bd	0.002	0.002	Bd	0.001	0.005	0.0027	Bd	Bd	Bd	Bd	Bd	Bd	Bd	Bd	Bd
**Zn**	0.009	0.038	0.015	0.023	0.004	0.025	0.012	0.0075	Bd	Bd	Bd	Bd	0.01	0.02	0.01	Bd	0.011	0.035	0.018	Bd	0.001	0.029	0.0075	0.002
**Hg**	Bd	Bd	Bd	Bd	Bd	Bd	Bd	Bd	Bd	Bd	Bd	Bd	Bd	Bd	Bd	Bd	Bd	Bd	Bd	Bd	Bd	Bd	Bd	Bd
**Be**	Bd	Bd	Bd	Bd	Bd	Bd	Bd	Bd	Bd	Bd	Bd	Bd	Bd	Bd	Bd	Bd	Bd	Bd	Bd	Bd	Bd	0.001	Bd	Bd
**V**	0.003	0.007	0.004	0.004	0.004	0.01	0.006	0.005	0.003	0.004	0.004	0.003	0.004	0.005	0.004	0.004	0.003	0.008	0.0045	0.003	0.004	0.005	0.004	0.004
**B**	4.22	4.57	4.4	4.45	4.16	4.62	4.4	4.21	4.41	4.6	4.53	4.53	4.42	4.86	4.59	4.68	4.55	4.8	4.71	4.63	3.99	4.57	4.36	4.29
**Co**	Bd	Bd	Bd	Bd	Bd	Bd	Bd	Bd	Bd	Bd	Bd	Bd	Bd	Bd	Bd	Bd	Bd	0.001	0.001	Bd	Bd	0.002	Bd	Bd
**Mo**	0.008	0.01	0.0089	0.008	0.01	0.013	0.011	0.012	0.009	0.013	0.011	0.01	0.006	0.01	0.0083	0.009	0.008	0.01	0.0092	0.009	0.008	0.01	0.0084	0.008
**Sb**	Bd	0.001	0.001	0.001	Bd	0.001	0.001	Bd	Bd	0.001	0.001	0.001	Bd	0.001	0.001	Bd	Bd	Bd	Bd	Bd	Bd	Bd	Bd	Bd
**Ba**	0.007	0.008	0.0075	0.008	0.008	0.015	0.011	0.008	0.006	0.008	0.007	0.006	0.009	0.013	0.0092	0.005	0.01	0.011	0.011	0.011	0.005	0.029	0.0093	0.007
**Bi**	Bd	Bd	Bd	Bd	Bd	Bd	Bd	Bd	Bd	Bd	Bd	Bd	Bd	Bd	Bd	Bd	Bd	Bd	Bd	Bd	Bd	Bd	Bd	Bd
**Sn**	Bd	Bd	Bd	Bd	Bd	Bd	Bd	Bd	Bd	0.001	0.001	Bd	Bd	0.001	0.001	0.001	0.002	0.006	0.0032	0.0045	0.001	0.001	0.001	0.001

Bd- Below detection, Bg- Background.

The presence of excess concentration of dissolved Cu and Fe in port Yamba water clearly indicates the impacts of trade with livestock, explosive products and organic waste from recreational boating and fishing fleet. The intensive activities of fishing fleet, break bulk and machinery and equipment from oil and gas refinery and preservative chemicals from the export of wood chips at port Eden are likely contributors to high concentrations of Zn, Mn, Fe, Cd and Co in the port water.

However, presence of excessive amount of Cu, Fe, Pb and Zn in port Botany confirm the impacts of the port activities which are associated with trade of cruide oil, fossil fuel, chemicals and bio-fuels. Moreover, the trade of coal, steel products, fertilizers, mineral sands and preservative chemicals from woodchips in port Newcastle signify the excess amounts of Cu, Fe, Zn, Mn in the port water. Finally, effluent from shipping activities, storage of hazardous products in the port vicinity are often overlooked [13] while they may be also the sources of metals in the studied port areas.

The variation of heavy metals between the different locations by means of ANOVA was also found insignificant, as shown in Table 4.

**Table 4:** Significance analysis in metal concentrations between background and port water.

Study Area	F		Df	P
**Port Jackson**	0.00024		36	0.9877
**Port Botany**	0.00092		36	0.9758
**Port Kembla**	0.00001648		36	0.9967
**Port Newcastle**	0.00007617		36	0.993
**Port Yamba**	0.002448		36	0.9608
**Port Eden**	0.01649		36	0.8985

Table 5 presents correlation between the water quality parameters and concentration of trace elements. Significant correlation at 5% significance (P < .05) was observed between Al, Pb, V, Cu and TDS; Cu, Fe, Zn, As and turbidity; Al, Fe, Mn, Zn, Fe and Al, Cu, Zn, Mn; and Cu, Zn, Fe, Pb and As, V, turbidity. Ba showed significant correlation with Fe and Al whereas Sb showed strong negative correlations with most of the trace metals and TDS. Only B was strongly correlated with pH, while Mo did not significantly correlate with any parameter.

**Table 5:** Correlation matrix between elements (bold correlations are significant at P < .05).

* *	pH	TDS	Turbidity	Conductivity	Al	As	Cu	Fe	Mn	Pb	Zn	V	B	Mo	Sb	Ba
**pH**	1															
**TDS**	-0.3448	1														
**Turbidity**	-0.7126	0.0857	1													
**Conductivity**	0.5613	0.5419	-0.5606	1												
**Al**	-0.5158	0.8905*	0.4892	0.3291	1											
**As**	-0.3957	-0.363	0.8810*	-0.6584	0.0812	1										
**Cu**	-0.4146	0.9939*	0.0889	0.4764	0.8716*	-0.376	1									
**Fe**	-0.5847	0.8499	0.5879	0.1885	0.9784*	0.1794	0.8366*	1								
**Mn**	-0.0078	0.8804	0.07945	0.6909	0.8272	-0.242	0.8266*	0.7985*	1							
**Pb**	-0.5570	-0.360	0.8814*	-0.7553	0.0713	0.9621*	-0.348	0.1542	-0.366	1						
**Zn**	-0.4489	0.9487*	0.2071	0.3339	0.8491*	-0.250	0.9541*	0.8743*	0.8357*	-0.266	1					
**V**	-0.7304	0.0626	0.9735*	-0.5352	0.4824	0.8649*	0.0694	0.5428	0.0085	0.9068*	0.1238	1				
**B**	0.7632*	-0.053	-0.2507	0.4896	-0.097	-0.072	-0.144	-0.074	0.4028	-0.338	-0.028	-0.381	1			
**Mo**	-0.3635	-0.252	0.3564	-0.2998	0.0206	0.4242	-0.230	-0.071	-0.434	0.5941	-0.413	0.5552	-0.649	1		
**Sb**	0.2499	-0.990*	-0.0033	-0.6407	-0.869*	0.4218	-0.976*	-0.801*	-0.888*	0.4214	-0.898*	-0.257	0.0179	0.2150	1	
**Ba**	-0.4945	0.6029	0.7747	0.0438	0.8706*	0.4984	0.5671	0.9209*	0.6904	0.4136	0.6566	0.7126	0.0982	-0.0070	-0.552	1

“*” denotes significant correlation value

Table 6 presents the results of the water quality indices based on trace metals used to assess the quality of the port water and to compare with different countries’ standards and with the background water. According to ANZECC, US- EPA and UK guidelines, the contamination index (C_d_) was high almost for all ports, except for some points at Port Botany, Port Kembla and Newcastle. However, Table 6 shows that sampling point 2 of Port Botany had low contamination index with respect to ANZECC standards but high contamination index according to the USEPA and UK standards. Similarly, sampling points 1, 2, 4 and 5 at Port Kembla and all sampling points at port Newcastle showed variations in contamination index according to different country standards. The background sample of Port Kembla was low and medium contaminated according to UK and ANZECC standards, while the other points showed variations in contamination according to different standards shown in Table 6. The background area of Port Kembla exhibited lower contamination than the other points. Almost all sampling points at Port Newcastle, including the background area, represented low and medium contamination according to ANZECC standards, whereas they depicted high contamination according to US EPA and UK standards, except for the background area that was medium contaminated during high tide. Port Yamba and Port Eden portrayed high contamination for all standard guidelines.

**Table 6:** Comparison of the contamination indices estimated based on the measured port water chemistry.

Study Area	Tide	Cd (ANZEEC)	Cd (EPA)	Cd (UK)	Cd Level	HEI (ANZEEC)	HEI (EPA)	HEI (UK)	HEI Level	WQI	Water Quality	TETI	EWQI
**Port Jackson background**	H	14.69	22.0949	70.94	HHH	21.77	28.0949	79.948	LLL	83.1	G	2021.5	0.041
	L	7.82	7.1	20.9	HHH	11.82	11.1	27.9	LLL	70.2	G	2024.15	0.034
**Port Jackson**	H	7.93–11.71	6.63–15.54	21.08–47.57	HHH	12.93–17.72	11.63–20.55	29.08–56.57	LLL	71.16–77.65	G	1931.2–2021.3	0.038–0.04
	L	6.52–7.96	5.82–13.11	16.87–55.97	HHH	11.65–14.61	9.82–22.85	23.87–63.97	LLL	71.04–87.15	G	1917.19–2072.5	0.036–0.041
**Port Botany background**	H	4.36	2.07	9.55	HMH	8.36	5.07	16.55	LLL	70.02	G	1922	0.036
	L	6.95	3.99	15.07	HHH	12.95	6.99	23.07	LLL	82.45	G	2078	0.039
**Port Botany**	H	0.27–13.46	4.89–21.67	18.55–72.07	LHH-HHH	4.73–17.46	7.89–25.68	25.55–80.7	LLL	71.15–76.70	G	1953.7–2096	0.034–0.039
	L	3.85–30.35	14.5–168.61	48.11–526.25	HHH	7.85–17.34	17.5–172.61	55.27–535.25	LLL-LLM	69.45–81.81	M*-G	1923.8–2443	0.030–0.042
**Port Kembla background**	H	3.094	-2.46	-2.73	HLL	9.094	1.54	5.263	LLL	74.4	G	2090.5	0.035
	L	1.83	4.48	-2.19	MHL	3.83	5.48	17.8	LLL	76.01	G	2015.6	0.037
**Port Kembla**	H	1.81–12.75	0.9–8.84	6.34–30.97	MMH-HHH	3.81–17.75	3.87–11.84	12.35–37.97	LLL	72.86–77.96	G	1978–2021	0.036–0.039
	L	1.41–5.7	1.35–6.09	5.16–18.12	MHH-HHH	3.41–9.7	3.35–7.9	11.16–21.36	LLL	77.67–83.05	G	1970–2050.3	0.038–0.04
**Port Newcastle background**	H	-0.92	1.58	4.85	LMH	0.078	2.58	8.85	LLL	77.03	G	1996	0.036
	L	-0.92	4.15	15.09	LHH	0.078	6.15	19.09	LLL	72.76	G	2041	0.039
**Port Newcastle**	H	0.92–1.43	4.7–10.29	13.85–31.03	LMH-MMH	0.078–3.54	6.13–11.9	19.85–36.04	LLL	74.25–78.88	G	2012–2045	0.035–0.039
	L	1.41–16.6	4–59.46	16.67–187.6	MHH-HHH	3.41–19.6	7–62.46	22.67–192.8	LLL	74.44–78.55	G	2024–2165	0.034–0.038
**Port Yamba background**	H	33.29	40.06	136.55	HHH	41.29	46.06	143.54	LLL	81.52	G	2041	0.039
	L	19.61	20.37	75.01	HHH	27.61	26.37	82.01	LLL	79.53	G	2135	0.037
**Port Yamba**	H	50.39–101.59	18.95–54.05	72.53–183.1	HHH	58.39–110.58	24.95–61.05	79.53–191.1	LLL	72.08–83.41	G	2131–2236	0.033–0.039
	L	48-67-220.43	44.41–594.5	162.6–1859	HHH	56.67–229.43	51.42–601.5	170.6–1867	LMH-LLL	73.11–74.66	G	2229–3002	0.024–0.033
**Port Eden background**	H	10.05	-5.92	-6.15	HLL	19.056	0.076	0.846	LLL	73.54	G	1914	0.038
	L	49.554	4.133	25.68	HHH	58.55	11.133	33.688	LLL	75.5	G	1897	0.039
**Port Eden**	H	2.17–10.5	5.92–5.96	6.10–6.16	LLL-HLL	2.66–6.82	0.033–0.074	0.09–0.89	LLL	70.6–76.46	G	1932–2019	0.037–0.038
	L	0.55–316.99	4.13–158.3	6.11–502.2	LLL-HHH	9.95–326	0.048–165.3	0.89–510.2	LLM-LLL	47.62–82.21	M*-G	1914–2006	0.024–0.042

H = High contaminated, M = Medium contaminated, L = Low contaminated, G = Good water quality, M* = Moderate water quality.

Cd = contamination index, HEI = heavy metal evaluation index, WQI-water quality index, TETI = trace element toxicity index, EWQI = environmental water quality index

The heavy metal evaluation index (HEI) shows that all sampling points at all ports were at low pollution, except for points 3 at Port Botany and Yamba and point 1 at Port Eden, which were classified as medium and high-polluted areas during low tide, according to the UK standard (Table 6). These are the same areas, which were classified as highly polluted according to the C_d_. The other points of Port Botany, Kembla, Yamba and Eden, classified as high and medium contaminated according to C_d_, were calculated as lower level pollution according to HEI.

The results inform that the various indices do not consistently predict the impact of various port activities on the water environment. The indices are designed to consider different attributes in the water quality for water quality assessment. Even though the WQI considers wider impacts of pollution on the water quality, it neglects the toxicity of metals present in the water. Likewise, C_d_ and HEI do not consider the toxicological impacts of the nutrients, physicochemical and biological parameters. To overcome this problem a separate new index based on the elemental toxicological impact, termed as trace element toxicity index (TETI), is used in this work. Table 6 gives the individual toxicological estimates for the elements based on their concentrations measured in each location and the relative toxicological weight. TETI indicates that boron had the highest impact on the toxicological profiles of all studied areas followed by aluminum, zinc, barium, arsenic, manganese, molybdenum, copper, vanadium, cobalt and lead. In addition to these findings, Port Jackson had high concentration of selenium, whereas cadmium and chromium were present only in the water of Port Yamba and Eden. Furthermore, TETI results state that Port Yamba had the highest index value followed by Port Botany, Newcastle, Kembla, Jackson and Port Eden. Additionally, in almost all ports the TETI value was high in the middle of the port area in comparison to the other points and the background area.

The proposed TETI only considers toxic elements in the water, while it overlooks the other fundamental water quality parameters used for WQI calculation. To overcome the gap of various index parameters, Environmental Water Quality Index (EWQI) is introduced that incorporates WQI and TETI indices. The EWQI clearly represents the impact of port activities on the water environment where the higher EWQI value represents better quality. Table 6 states that all ports have similar EWQI values, except for some points at Port Botany, Yamba and Eden, which have lower values unveiling the comparative bad water quality of those ports.

Evaluation of the four indices used to determine pollution level reveals the important contaminants and anthropogenic inputs of metals and other pollutants in the study areas. Although all four indices specify varying levels of contamination in the studied areas their outcomes are not uniform. This is because each index considers different pollutants of importance for their calculated results. For instance, WQI index considers nine parameters which are physicochemical, nutrients and one biological parameter and disregards the toxicity of metals in the aquatic system. Based on the WQI only one case for port Botany and three cases for port Eden during low tide exhibited medium water quality, mainly due to high turbidity, low DO and fecal coliforms, while for all of the other sampling sites the water quality was good. Both HEI and C_d_ disregard the physicochemical and biological impacts on water quality and consider the heavy metals or hydrocarbons in relation to the recommended national guideline threshold values. In this study the HEI index showed that one sampling site at port Botany, Eden and Yamba had water quality of medium contamination and one site in port Yamba of high contamination. In all cases this was a result of significant iron concentrations and relative to the UK and US EPA guidelines. The C_d_ index in most showed high contamination due to either the copper content relative to the AZECC guidelines or iron content relative to the US EPA and UK guidelines. These findings clearly showcase the limitations of each index and also limitations of the international water quality guidelines, which are, firstly non-standardized between different countries and, secondly, do not provide guidelines for a number of pollutants. The newly established EWQI attempts to overcome the limitations of the current water assessment indices and considers all pollutants ranging from physicochemical, biological and the individual toxicity levels of each trace element. The EWQI index in this study presented that the trace element of most importance of the water quality in the studied ports is boron, as shown in S4 Table. Boron appears in high concentrations in the water samples, has total score of 438 according to the ATSDR [37] assessment, but is not considered in any of the international guidelines, hence it is not accounted for in either of the WQI, C_d_ or HEI indices.

## Conclusions

This study examines the extent of physiochemical and biological constituents present in the port water. The extensive study on pollutants of different port land uses and the comparison with the respective background area advocates a number of important considerations in the port water environment in NSW, Australia. The water quality index (WQI) analysis of the port area unveiled that the lower DO levels, higher turbidity and fecal coliforms markedly reduced the water quality of Port Botany and Eden. The trace metal concentrations in the port water provide baseline information for understanding the pollution levels, such as the high concentrations of copper at Port Jackson, high concentrations of copper and lead at Port Botany and high concentrations of copper and zinc at Port Kembla. Furthermore, Port Newcastle had high concentration of copper and manganese; Port Yamba was enriched with copper, manganese and lead, while port Eden had very high concentrations of copper, manganese, cadmium and cobalt. Contamination index and heavy metal evaluation index also revealed the level of contamination and heavy metal index. The contamination index preseneds high contamination levels in all of the studied ports areas. In addition, the heavy metal evaluation index depicted Port Botany, Yamba and Eden as high and medium polluted areas. Different water quality indices used in this study, apply different water quality indicators that help assess the overall water quality of the port area. WQI considers physicochemical, biological and nutrients but overlooks the toxicological indicators whereas; C_d_ and HEI consider only the toxicological parameters neglecting the physicochemical, biological and nutrient pollution. The study further explains the EWQI, which incorporates all the physicochemical, biological and toxicological indicators to assess the quality of the port water in a more comprehensive way. This research work points out that on an average the quality of the port water is good, except for Port Botany, Yamba and Eden, and recommends regular monitoring and management of port activities accounting for both biological and chemical toxicological profiles of the discharging activities.

## Supporting information

S1 Table Sample site identification. (DOCX)

S2 Table Australian and international standards and guidelines for marine water ecosystem. (DOCX)

S3 Table Original data for water quality parameters. (XLSX)

S4 Table Calculation data for Environmental Water Quality Index (EWQI). (XLSX)
