# Supplementary material for: Water quality assessment of Australian ports using water quality evaluation indices
Source: PLoS One. 2017 Dec 15;12(12):e0189284. doi: 10.1371/journal.pone.0189284 (PMC5731693; doi:10.1371/journal.pone.0189284)
Supplement: S1 Table — (DOCX) [file pone.0189284.s001.docx]

Table S1. Sample site identification.

| Study area | Site Id | Coordinates | Study area | Site Id | Coordinates |
| --- | --- | --- | --- | --- | --- |
| Port Jackson | 1 | S 33.853570, E 151.208512 | Port Newcastle | 1 | S 32.925416, E 151.778577 |
|  | 2 | S 33.859807, E 151.209741 |  | 2 | S 32.925539, E 151.782635 |
|  | 3 | S 33. 859842, E 151.212707 |  | 3 | S 32.924978, E 151.786634 |
|  | 4 | S 33.857741, E 151.215940 |  | 4 | S 32.923327, E 151.790202 |
|  | 5 | S 33.859411, E 151.221616 |  | 5 | S 32.924313, E 151.793066 |
| Port Botany | 1 | S 33.954812, E 151.193165 | Port Yamba | 1 | S 29.431120, E 153.341446 |
|  | 2 | S 33.957034, E 151.196641 |  | 2 | S 29.432980, E 153.342746 |
|  | 3 | S 33.958707, E 151.197874 |  | 3 | S 29.433824, E 153.343594 |
|  | 4 | S 33.976960, E 151.226426 |  | 4 | S 29.435538, E 153.345395 |
|  | 5 | S 33.979771, E 151.228915 |  | 5 | S 29.434685, E 153.346981 |
| Port Kembla | 1 | S 34.437268, E 150.902012 | Port Eden | 1 | S 37.072537, E 149.909572 |
|  | 2 | S 34.445780, E 150.900463 |  | 2 | S 37.072786, E 149.907408 |
|  | 3 | S 34.463832, E 150.896941 |  | 3 | S 37.071524, E 149.907529 |
|  | 4 | S 34.464195, E 150.898728 |  | 4 | S 37.071228, E 149.905944 |
|  | 5 | S 34.465885, E 150.900251 |  | 5 | S 37.074487, E 149.910094 |
