# Supplementary material for: Water quality assessment of Australian ports using water quality evaluation indices
Source: PLoS One. 2017 Dec 15;12(12):e0189284. doi: 10.1371/journal.pone.0189284 (PMC5731693; doi:10.1371/journal.pone.0189284)
Supplement: S2 Table — (DOCX) [file pone.0189284.s002.docx]

Table S2. Australian and international standards and /or guidelines for Marine water ecosystem.

| Parameter (mg/l) | ANZECC, 2000 (Marine water trigger value) | USEPA, 2009  (MCL) | Uk, 2014  (MAC) |
| --- | --- | --- | --- |
| pH | 8.4 |  |  |
| DO % | 90 |  |  |
| Fecal Coliform (MPN) |  | 9.9 |  |
| Silver | 0.0008 | - | - |
| Aluminum | - |  |  |
| Arsenic | - | 0.036 | 0.025 |
| Cadmium | 0.0007 | 0.0093 |  |
| Chromium | 0.0077 | - | 0.015 |
| Copper | 0.0003 | 0.0031 | 0.00376 |
| Iron | - |  | 0.001 |
| Manganese | - |  | - |
| Nickel | 0.007 | 0.0082 | 0.03 |
| Lead | 0.0022 | 0.0081 | 0.025 |
| Selenium | - | 0.071 | - |
| Zinc | 0.007 | 0.081 | 0.04 |
| Mercury | 0.0001 | 0.00094 |  |
| Beryllium | - | - | - |
| Vanadium | 0.05 |  | 0.1 |
| Boron | - | - | 7 |
| Cobalt | 0.000005 | - | - |

(ANZECC, 2000); (USEPA, 2009); (DEFRA, 2014)

MAC = Maximum admissible concentration; MCL = Maximum concentration limit
